# Plasma proteome profiling reveals biomarkers of chemotherapy resistance in patients with advanced colorectal cancer

**DOI:** 10.1002/qub2.34

**Published:** 2024-02-14

**Authors:** Jingxin Yang, Jin Chen, Luobin Zhang, Fangming Zhou, Xiaozhen Cui, Ruijun Tian, Ruilian Xu

**Affiliations:** ^1^ Medical Genetic Center Affiliated Shenzhen Maternity and Child Healthcare Hospital Southern Medical University Shenzhen China; ^2^ Clinical Center for Molecular Diagnosis and Therapy The Second Affiliated Hospital of Fujian Medical University Quanzhou China; ^3^ Department of Chemistry and Research Center for Chemical Biology and Omics Analysis School of Science Southern University of Science and Technology Shenzhen China; ^4^ Department of Oncology Shenzhen People’s Hospital (The Second Clinical Medical College, Jinan University; The First Affiliated Hospital, Southern University of Science and Technology) Shenzhen China

**Keywords:** biomarker, chemotherapy resistance, colorectal cancer, plasma proteome

## Abstract

Colorectal cancer (CRC) is one of the most common cancers. Patients with advanced CRC can only rely on chemotherapy to improve outcomes. However, primary drug resistance frequently occurs and is difficult to predict. Changes in plasma protein composition have shown potential in clinical diagnosis. Thus, it is urgent to identify potential protein biomarkers for primary resistance to chemotherapy for patients with CRC. Automatic sample preparation and high‐throughput analysis were used to explore potential plasma protein biomarkers. Drug susceptibility testing of circulating tumor cells (CTCs) has been investigated, and the relationship between their values and protein expressions has been discussed. In addition, the differential proteins in different chemotherapy outcomes have been analyzed. Finally, the potential biomarkers have been detected via enzyme‐linked immunosorbent assay (ELISA). Plasma proteome of 60 CRC patients were profiled. The correlation between plasma protein levels and the results of drug susceptibility testing of CTCs was performed, and 85 proteins showed a significant positive or negative correlation with chemotherapy resistance. Forty‐four CRC patients were then divided into three groups according to their chemotherapy outcomes (objective response, stable disease, and progressive disease), and 37 differential proteins were found to be related to chemotherapy resistance. The overlapping proteins were further investigated in an additional group of 79 patients using ELISA. Protein levels of F5 and PROZ significantly increased in the progressive disease group compared to other outcome groups. Our study indicated that F5 and PROZ proteins could represent potential biomarkers of resistance to chemotherapy in advanced CRC patients.

**Abbreviations**
5‐FU: 5‐fluorouracil
APC: activated protein C
CRC: colorectal cancer
CTCs: circulating tumor cells
ctDNA: circulating tumor DNA
CV: coefficient of variation
DPD: dihydropyrimidine dehydrogenase
ELISA: enzyme‐linked immunosorbent assay
FX: coagulation factor X
GMR: glucose metabolic rate
LFQ: label‐free quantification
MS: mass spectrometry
OR: objective response
PD: progressive disease
QC: quality control
SD: stable disease
TP: tyrosine phosphorylase
TS: thymidylate synthase
TTP: time to progression
ZPI: protein Z‐dependent protein inhibitor

## 1 INTRODUCTION

Colorectal cancer (CRC) has the third highest cancer incidence in the world. In 2020, there were 1.93 million new patients with CRC accounting for 10% of new cancer patients [1]. The mortality rate due to CRC is the second leading cause of death from malignant tumors in the world [1] and is the fifth highest in China. The treatment approaches used for patients with CRC are grim [2]. Approximately 25%–30% of CRC patients present liver metastases and only 25% are amenable to surgical resection, which is considered the main treatment [3]. Most CRC patients are diagnosed in stages III or IV, and those ineligible for surgical resection can only rely on treatment based primarily on chemotherapy. There are two types of chemoresistance defined according to the time‐to‐progression (TTP) of tumor patients: primary drug resistance and secondary drug resistance. Although the FOLFOX chemotherapy regimen (consisting of 5‐fluorouracil, leucovorin calcium, and oxaliplatin) is considered the gold standard treatment, over half of CRC patients still have no response to this chemotherapy regimen [4, 5, 6]. That is, primary drug resistance frequently occurs. It is difficult to predict chemotherapy results in CRC patients, which include objective response (OR), stable disease (SD), and progressive disease (PD). This has led to enormous mental and physical strain and economic pressure among patients and has led to a waste of medical resources. Thus, it is urgent to identify potential biomarkers for primary resistance to chemotherapy drug for patients with CRC.

Human plasma is extremely valuable in clinical and biological research because it can reflect the physiological and pathological status of patients [7]. It contains several types of biomarkers, and proteins have been identified as biomarkers for the diagnosis and prognosis of CRC [8]. In addition, circulating tumor DNA (ctDNA) can predict recurrence‐free survival and the effects of adjuvant chemotherapy [9]. Circulating tumor cells (CTCs) in human plasma reflect tumor metastasis [10], and their drug susceptibility testing indicates a patient’s response to chemotherapy. The drug susceptibility testing of CTCs is strongly related to clinical outcomes in lung cancer, which means that it may guide clinical treatment and avoid primary drug resistance [11]. Furthermore, our previous work demonstrated that CTCs chemosensitivity tests could accurately assess the clinical response in multiple advanced cancers [12]. Therefore, CTCs drug susceptibility testing could predict the potential outcomes of chemotherapy, sensitivity or resistance, and its relationship to plasma proteins may contribute to identify protein biomarkers for chemotherapy resistance.

Mass spectrometry (MS)‐based proteomics is a powerful tool for the discovery of plasma biomarkers. Due to the automatic and integrated preparation and state‐of‐the‐art LC‐MS/MS, a high throughput and high‐sensitivity analysis of plasma proteins is possible [13]. Plasma protein profiles have been used for the diagnosis and prognosis of alcohol‐related liver disease [14]. In a multiomics study of lung adenocarcinoma, the plasma proteome was used to discover potential biomarkers and explain the relationship between genome abnormalities and carcinogenic proteins [15]. In our previous work, we applied automatic sample preparation to clinical plasma samples and developed a rapid, robust, and highly reproducible workflow [16]. Therefore, it may be feasible to easily distinguish patients with different chemotherapy outcomes (PD, SD, and OR) through plasma protein profiling, which would contribute to discovering biomarkers as indicators of chemotherapy resistance.

In this study, plasma proteins from 60 patients with advanced CRC were profiled, and their correlations with the results of CTCs chemosensitivity testing were evaluated. Plasma proteins were also used to distinguish chemotherapy outcomes (OR, PD, and SD). According to these two strategies, proteins F5 and PROZ in plasma were found to be upregulated in patients resistant to chemotherapy. These protein patterns were then verified in 79 CRC patients by enzyme‐linked immunosorbent assay (ELISA). Thus, F5 and PROZ could predict chemotherapy resistance in patients with advanced CRC.

## 2 RESULTS

### 2.1 Automatic preparation is robust and highly reproducible

**FIGURE 1:**
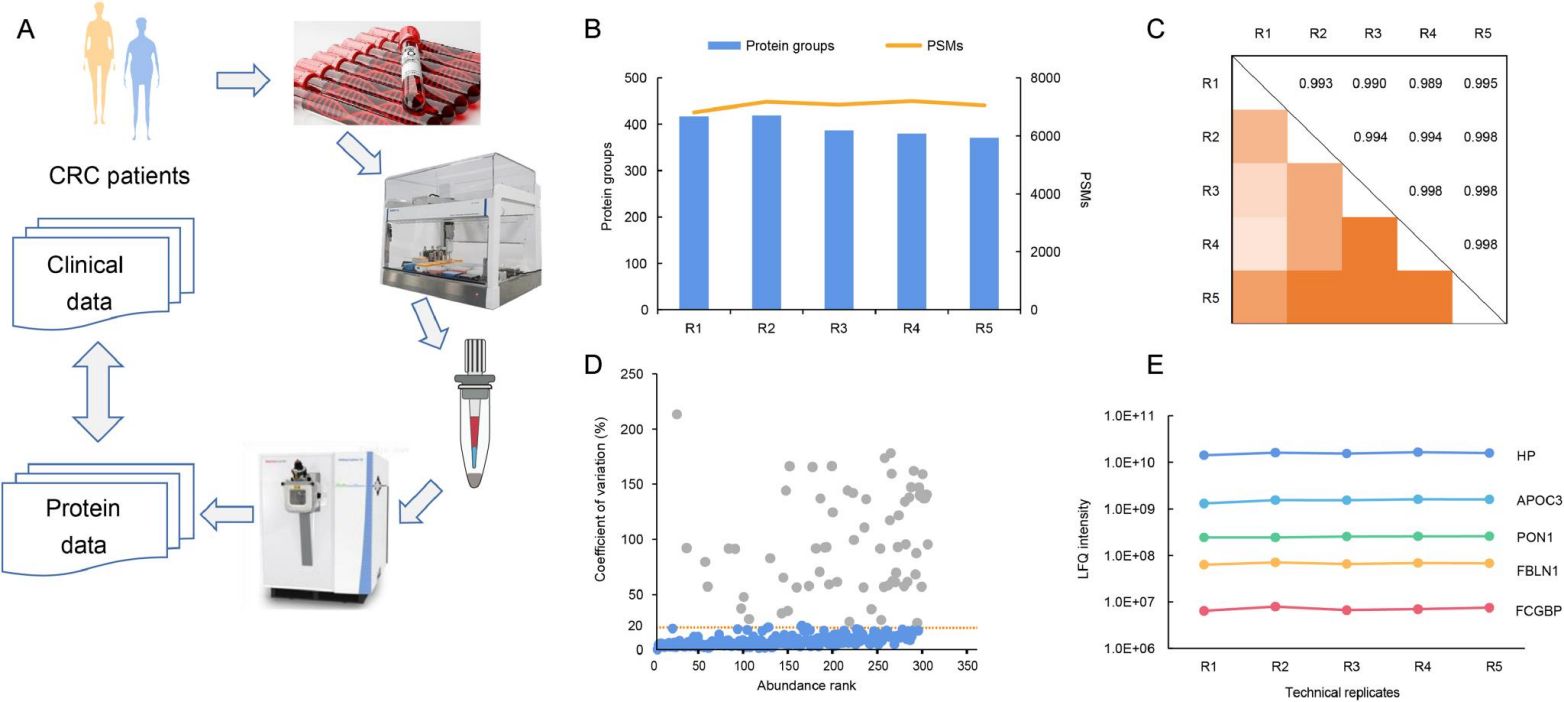
Detection of plasma proteins based on automatic preparation. (A) Schematic representation of the sample workflow. (B) Protein groups and PSMs of five technical workflow replicates. (C) Color‐coded Pearson correlation coefficient of five replicates in the technical workflow. (D) CVs of all quantified proteins calculated from the five technical workflow replicates. (E) Reproducibility of the LFQ intensities of five proteins covering nearly five orders of magnitude for five technical workflow replicates. CVs, coefficients of variation; LFQ, label free quantification.

We attempted to associate data from the plasma proteome with clinical data of patients with advanced CRC cancer (Figure 1A). Briefly, plasma from patients with CRC was collected, and the preparation of 32 samples was simultaneously automated, including steps of plasma dilution, cysteine carbamidomethylation, and enzyme digestion. The desalted samples were analyzed using nanoLC‐MS/MS. In our previous study, we found that automated plasma preparation saved time and labor, and microflow LC‐MS/MS showed high reproducibility [16]. In our workflow, plasma protein analysis is robust and highly reproducible. In five technical workflow replicates, the number of protein groups and PSMs were 395 ± 22 and 7059 ± 155, respectively, which were basically consistent for each replicate (Figure 1B). In addition, we quantified the plasma proteins (Table S1). The Pearson correlation coefficients obtained ranged from 0.989 to 0.998 (Figure 1C). The coefficients of variation (CVs) of five technical workflow replicates showed that 76.5% were below 20% with a median value of 8% (Figure 1D). Furthermore, we selected five proteins from five technical workflow replicates that could cover five orders of magnitude and found that the measurements and hence workflow with automatic preparation were robust and highly reproducible (Figure 1E).

### 2.2 Plasma proteins were significantly correlated with drug resistance based on drug susceptibility testing of CTCs

**FIGURE 2:**
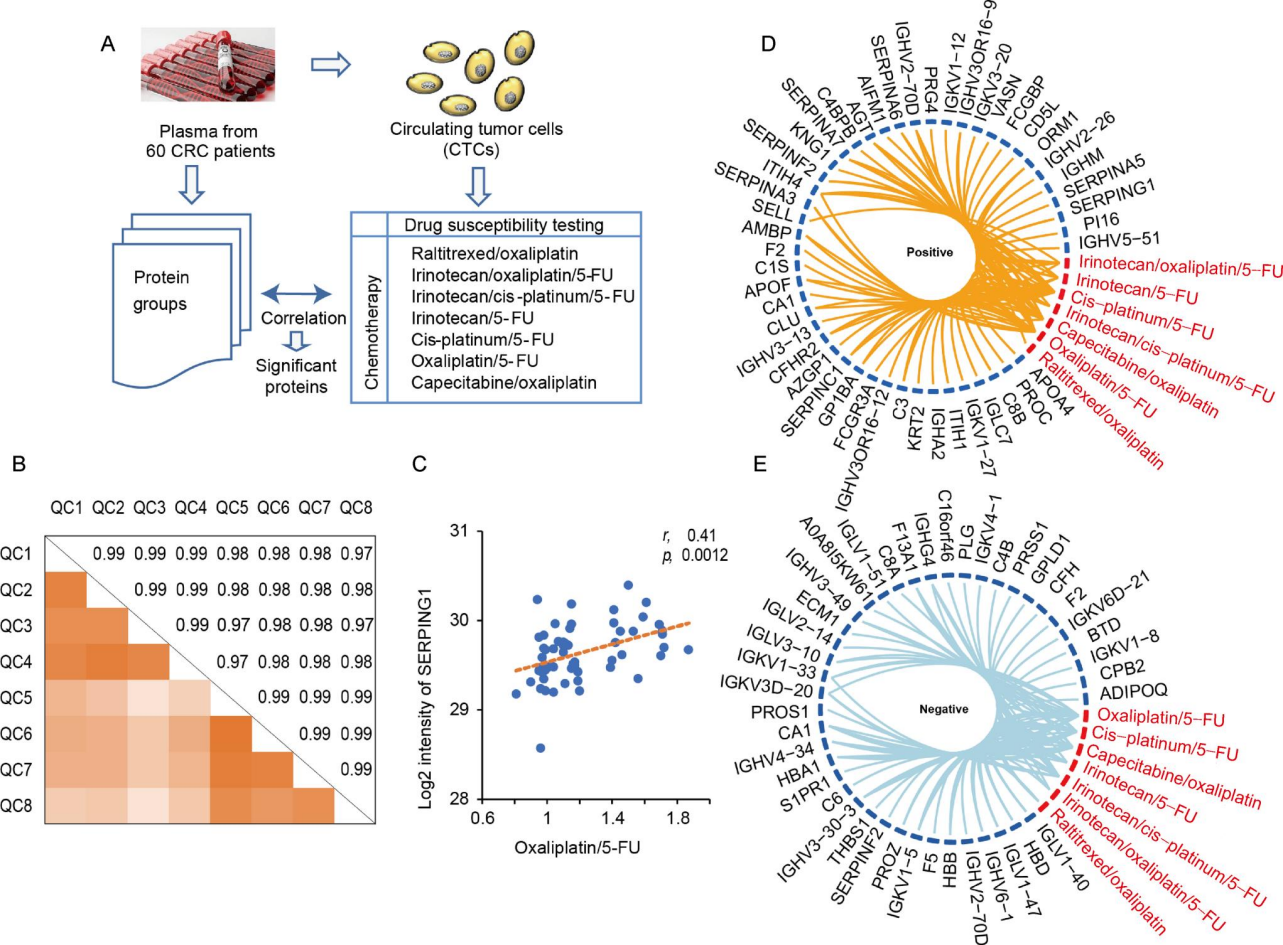
Drug susceptibility testing of CTCs and their correlation with plasma proteins. (A) Schematic representation of the workflow. (B) Color‐coded Pearson correlation coefficient for binary comparison of QC samples during detection of 60 plasmas from CRC patients. (C) The correlation between SERPING1 protein intensity and drug susceptibility values when treated with oxaliplatin/5‐FU. (D, E) The positive correlation (D) and the negative correlation (E) of plasma proteins and drug resistance in different chemotherapy regimens. The red and black labels represent chemotherapy regimens and proteins, respectively, and the lines refer to their correlation. 5‐FU, 5‐ fluorouracil; CTCs, circulating tumor cells; CRC, colorectal cancer; QC, quality control.

The drug susceptibility testing of CTCs reflected therapeutic effects of different chemotherapies, and the correlations between these responses and plasma proteins were analyzed (Figure 2A). First, we collected plasma samples from 60 CRC patients (Table S2), and their proteins were quantified (Table S3). Quality control samples were run every 8–10 clinical samples and were used to evaluate the stability of MS detection. The Pearson correlation coefficient ranged from 0.97 to 0.99 (Figure 2B) indicating that the system was stable. In addition, we extracted the intensities of five proteins covering nearly five orders of magnitude and values showed limited changes (Figure S1A). The label free quantification (LFQ) intensity of all quantified proteins from the QC samples was essentially the same (Figure S1B). Thus, our strategy showed excellent reproducibility.

We then investigated the correlations between protein levels quantified by MS (Table S3), and the levels of chemosensitivity were detected using CTC’s drug susceptibility testing (Table S4). The Pearson correlation coefficients were calculated for each chemotherapy regimen. For example, the drug susceptibility values, when CTCs from 60 patients were treated with oxaliplatin combined with 5‐FU, were used to calculate their connection to the protein intensity of SERPING1 from the same patients detected using MS, and positive correlation with *p* = 0.0012 was observed (Figure 2C). Then, proteins with significant positive and negative correlations (*p* < 0.05) under different chemotherapy regimens were pooled. In addition, 47 and 42 proteins showed positive and negative correlations with drug resistance, respectively (Table S5, Figure 2D,E). The protein expression of CA1, F2, IGHV2‐70D, and SERPINF2 showed an opposite correlation with different chemotherapeutic agents, while the other proteins showed the same behavior when treated with different chemotherapeutics. Therefore, 85 proteins were collected irrespective of the type of chemotherapy, and they were used for further study.

### 2.3 Differential plasma proteins in CRC patients with different chemotherapy outcomes

**FIGURE 3:**
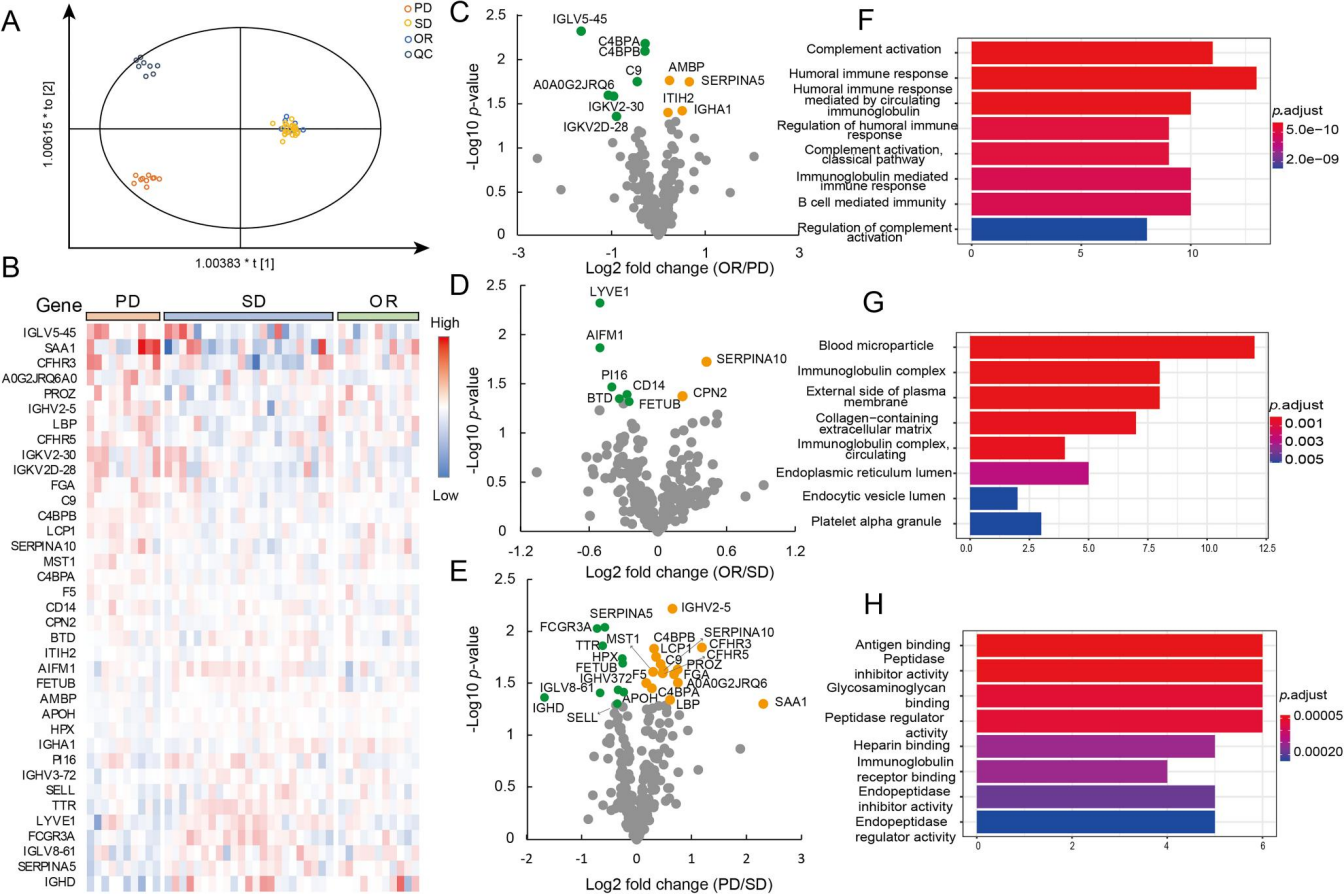
Differentially expressed proteins from CRC patients with different outcomes of chemotherapy. (A) OPLS‐DA model for classifying CRC patients with different chemotherapy outcomes. (B–E) Heatmap (B) and volcano (C–E) graphs in different chemotherapy outcomes. Proteins with significant changes were labeled with color in volcano graphs (orange for upregulation and green for downregulation). (F–H) Biological progress (F), cellular component (G), and molecular function (H) of 37 differentially expressed proteins. CRC, colorectal cancer.

Since parts of the selected CRC patients had not been evaluated for tumor response or the available clinical data were incomplete, we ultimately chose 44 samples for further analysis. These patients were divided into three groups according to their clinical outcomes: 11 OR patients, 23 SD patients, and 10 PD patients (Table 1 and Table S2). Their plasma protein expression profiles were obtained, and 44 samples were clustered using the OPLS‐DA model. In detail, the PD group could be clearly distinguished from the SD and the OR groups, while the SD and the OR groups were inseparable (Figure 3A). The QC samples were clustered, which indicated that our sample workflow was stable (Figure 3A). According to the screening criterion, ANOVA analysis showed a significant difference (*p* < 0.05); 37 proteins were found to be significantly changed and their heatmaps are shown (Figure 3B). Volcano graphs between OR and PD, OR and SD, as well as PD and SD are shown in Figure 3C–E. respectively, and significantly differential proteins were labeled. We also performed GO enrichment analysis to identify more information on the biological implications of these 37 differentially expressed proteins. The enrichment of the biological process showed that differentially expressed proteins participated in the activation of the complement and in immune response (Figure 3F). The enrichment of the cellular components demonstrated that changes in proteins occurred in blood microparticles, the immunoglobulin complex, the external side of the plasma membrane, and in the lumens (Figure 3G). The molecular functions focused on binding and peptidase activity (Figure 3H). Furthermore, the analysis of the KEGG pathway described differential proteins enriched in complement and coagulation cascades.

**TABLE 1:** Test cohort with 44 colorectal cancer patients enrolled.

Type	Progressive disease	Stable disease	Objective response
Sample size	10	23	11
Age (average ± SD)	60 ± 6	58 ± 7	62 ± 7
Sex (%, male)	70	70	45

### 2.4 F5 and PROZ levels were markedly increased in patients with chemotherapy resistance

**FIGURE 4:**
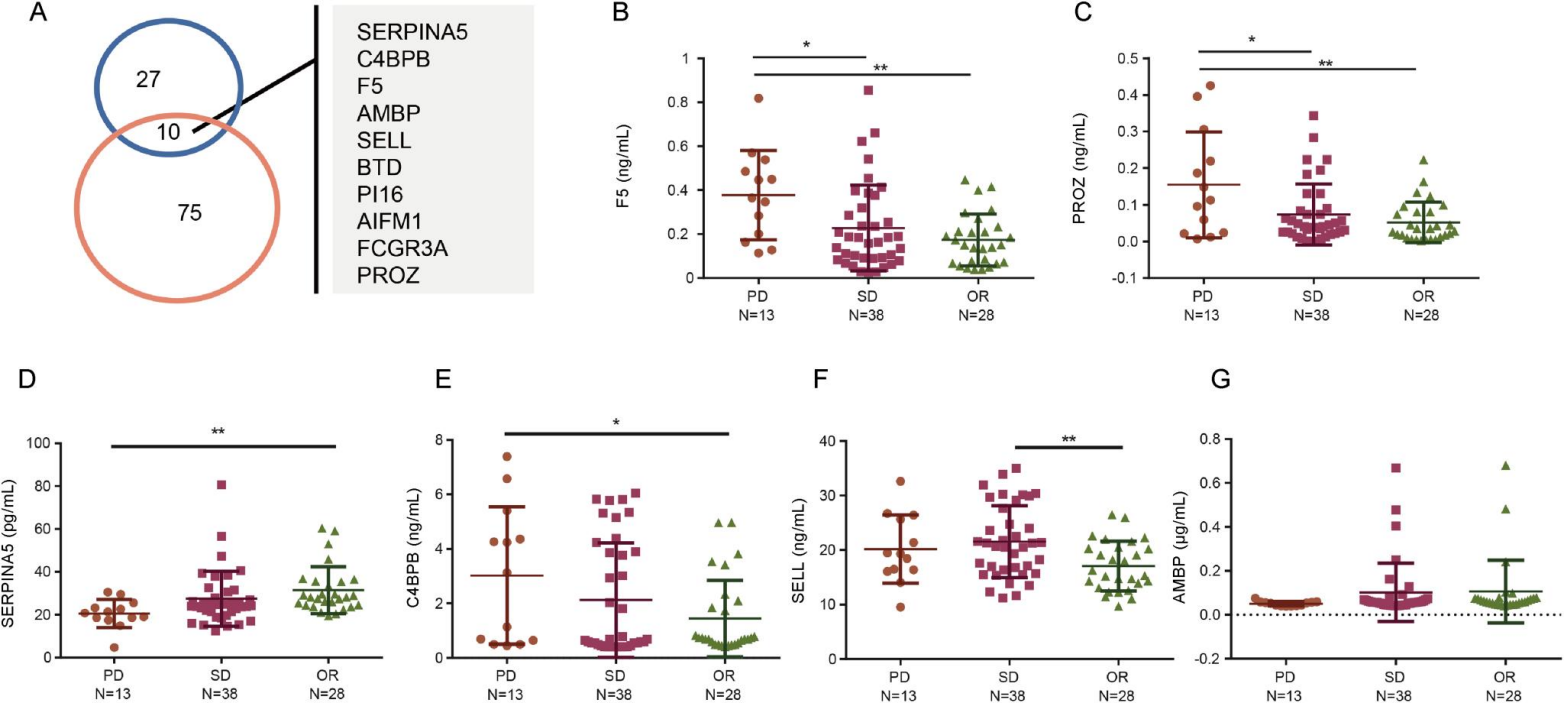
The expression of significantly differential proteins in different chemotherapy outcomes detected by ELISA. (A) The overlapping proteins based on two strategies. (B–G) Expression of F5 (B), PROZ (C), SERPINA5 (D), C4BPB (E), SELL (F), and AMBP (G) detected using ELISA. ELISA, enzyme‐linked immunosorbent assay.

The above two strategies identified 85 and 37 differentially expressed proteins associated with chemotherapy resistance and ten proteins overlapped (Figure 4A). We conducted a PubMed search to determine whether these proteins had already been reported to be related to cancer. F5 was found to participate in the coagulation cascade reaction and to serve as a prognostic biomarker for gastric cancer [17]. PROZ is an anticoagulation factor, and its serum level is higher in patients with pancreatic cancer than in patients with benign pancreatic tumors [18]. SERPINA5 is an inhibitor of activated protein C (APC) and may participate in tumor inhibition [19]. C4BPB can inactivate protein S which is a cofactor to APC [20]. AMBP can be hydrolyzed into two functional proteins, *α*‐1‐microglobulin and bikunin, which participate in many biological processes [21]. SELL (L‐selectin) is a type of selectin, which can promote the interaction between cancer cells and blood components (platelets, endothelial cells, and white blood cells), while increased selectin ligands in cancer cells have been associated with cancer metastasis and poor prognosis [22]. In addition, we investigated the performance of six differential proteins in the QC sample to evaluate their stability under MS detection (Figure S2). Their CVs were below 20% except for those of PROZ (36%) and SERPINA5 (23%). Therefore, we chose six differential proteins (SERPINA5, C4BPB, F5, AMBP, SELL, and PROZ) for further study, which had been reported as potential biomarkers related to cancers [17, 18, 21, 23, 24, 25].

In total, 79 CRC patients at stages III or IV were identified and served as a validation cohort. Patients were divided into three groups according to their tumor responses (13 PD responders, 38 SD responders, and 28 OR responders) (Table 2 and Table S6). Their plasma samples were collected, and the plasma levels of SERPINA5, C4BPB, F5, AMBP, SELL, and PROZ were measured by ELISA. The results revealed that plasma F5 and PROZ concentrations increased in the PD group compared to the SD group (*p* = 0.0226 and *p* = 0.016, respectively) and the OR group (*p* = 0.0002 and *p* = 0.002, respectively) (Figure 4B,C). SERPINA5 and C4BPB protein levels decreased and increased in the PD group compared to the OR group (*p* = 0.0019 and *p* = 0.0136) (Figure 4D,E), respectively, while SELL and AMBP did not show any significant changes between PD outcomes and those of other groups (Figure 4F,G). Therefore, both F5 and PROZ protein levels were significantly upregulated in the PD group compared to the SD group and the OR group, which indicated that chemotherapy resistance could occur with the overexpression of F5 and PROZ proteins. Furthermore, the intensities of F5 and PROZ detected by LC‐MS/MS increased in the PD group compared to the SD group (*p* = 0.031 and *p* = 0.023, respectively, Figure S3A,B). Their fragments of peptides confirmed the changes in protein levels (Figure S3C,D). That is, F5 and PROZ showed similar behaviors with the plasma proteome and the ELISA method. Although the proteins did not show a significant change between the PD and OR groups according to MS intensity, this may be attributed to the small sample size. The behaviors of other potential markers (AMBP, SERPINA5, SELL, and C4BPB) and their fragments of peptides in the test cohort by LC‐MS/MS detection are shown in Figure S4. SERPINA5 and C4BPB showed significant downregulation and upregulation in the PD group compared with SD and OR groups, respectively (Figure S4A–D) but it showed significant changes only in the PD group compared with the OR group in the validation cohort. SELL showed significant changes between PD and SD groups in the test cohort (Figure S4E,F), but the differences could only be observed between SD and OR groups in the validation cohort. Although AMBP significantly decreased in the PD group compared with SD and OR groups with the method of LC‐MS/MS (Figure S4G,H), it could not be observed in the validation cohort. Altogether, these experiments showed that plasma protein levels of F5 and PROZ could be potential biomarkers for predicting primary drug resistance.

**TABLE 2:** Validation cohort with 79 colorectal cancer patients enrolled.

Type	Progressive disease	Stable disease	Objective response
Sample size	13	38	28
Age (average ± SD)	59 ± 5	58 ± 6	61 ± 6
Sex (%, male)	38	71	68

## 3 DISCUSSION

The most crucial problem of chemotherapy outcome is primary or secondary drug resistance [26]. Indeed, even the FOLFOX chemotherapy regimen has been associated with 50% of patients with primary drug resistance, and many studies have investigated the mechanisms responsible. For example, 5‐FU is a widely present uracil analog in various chemotherapy regimens and can produce a series of metabolites that interfere with the process of DNA replication [5] and RNA synthesis [27]. Its mechanism of drug resistance is related to the upregulation of thymidylate synthase (TS) expression [28, 29], the downregulation of tyrosine phosphorylase (TP) [30], and the high‐level expression of dihydropyrimidine dehydrogenase (DPD) [31, 32]. However, most of these studies relied on cell lines and may not be consistent with the actual clinical setting. Therefore, drug resistance research based on primary clinical samples is more helpful for identifying predictable biomarkers.

In this study, drug susceptibility testing of CTCs was used to evaluate tumor responses to treatment with chemotherapy contributing to a more targeted selection of chemotherapy treatment. That is, the results of drug susceptibility testing of CTCs could reflect drug resistance under different chemotherapy regimens. Therefore, any correlation between plasma protein levels and the results of drug susceptibility testing could provide data supporting potential biomarkers of primary drug resistance. We identified 85 proteins whose expressions were significantly correlated with chemotherapy resistance.

Using plasma to discover biomarkers based on quantitative proteomics has proven to be a useful method in cancer biomarker research. LC‐MS/MS analysis has been used in different clinical diagnoses protocols and is undoubtedly a routine technique [33]. In our study, patients with different chemotherapy outcomes (PD, OR, and SD) were clustered according to their plasma proteins levels. The PD group showed disease progression in response to chemotherapy indicating chemotherapy resistance. We attempted to identify significantly differentially expressed proteins between the PD group and other groups and found that F5 and PROZ were upregulated in both the test and validation cohorts when chemotherapy resistance occurred. Therefore, analysis of the plasma proteome is a powerful tool to discover potential biomarkers.

The two differentially expressed proteins F5 and PROZ identified in this study are both related to coagulation factor X (FX), which is the key factor in the coagulation cascade [34]. Many studies have shown that the activation of coagulation and fibrinolysis is closely related to the occurrence and development of cancer [17, 35]. F5 (coagulation factor V, 330 kDa) is a factor involved in the coagulation cascade reaction. It can form a complex with activated FX, Ca^2+^, and phospholipid and can convert prothrombin into thrombin and participate in coagulation [17]. In contrast to F5, the complex of PROZ and protein ZPI can inhibit the activation of FX and thus limit blood coagulation [36, 37, 38]. Interestingly, the inhibitors and activators of FX, F5, and PROZ were found to be increased in patients with PD using the method of MS‐based plasma proteome and ELISA. This may be due to the fact that under physiological conditions, the upregulation of coagulation factors is often accompanied by the increased expression of their inhibitors. The data obtained in this study showed that the intensities of F5 and PROZ in the PD group were upregulated suggesting a role for the overexpression of F5 and PROZ in chemotherapy resistance. Although F5 and PROZ are reported to be involved in cancer development, our work is the first to demonstrate that F5 and PROZ have the potential to predict primary drug resistance in CRC.

There were some limitations and weaknesses in this study. Firstly, our test cohort and validation cohort were not large enough. Secondly, the sex ratio of the patients included in this study was not strictly comparable, although sex differences in this study had little influence on the conclusion.

## 4 CONCLUSION

The plasma protein proteome of 60 patients with CRC was quantified using MS‐based profiling with automatic sample preparation and high‐throughput analysis. CTCs drug susceptibility testing and chemotherapy outcomes were used to evaluate primary drug resistance. According to these two strategies, chemotherapy resistance was found to be correlated with 85 and 37 proteins and 10 proteins overlapped. Finally, the levels of F5 and PROZ proteins were found to markedly increase in the PD group in both the test and the validation cohorts. Thus, F5 and PROZ might be considered potential biomarkers for chemotherapy resistance in patients with advanced CRC.

## 5 MATERIALS AND METHODS

### 5.1 Clinical samples

Plasma samples were obtained from the Department of Oncology of Shenzhen People’s Hospital (Guangdong, China). The study was approved by the ethical committee at the Shenzhen People’s Hospital, and the study was performed according to the specifications of the Declaration of Helsinki. A total of 139 plasma samples from patients with advanced CRC were collected, and their detailed clinical information was obtained (Tables S2 and S6). Sixty plasma samples were used to evaluate potential correlations between plasma protein levels and CTC susceptibility testing results (Table S2). To investigate the associations between plasma protein levels and chemotherapy outcomes, 44 plasma samples were used as a test cohort (Table 1 and Table S2) and 79 samples were used as a validation cohort (Table 2 and Table S6). Chemotherapy outcomes were evaluated by computed tomography and magnetic resonance imaging according to response evaluation criteria in solid tumors [39]. In summary, OR was defined as a decrease of at least 30% of the longest diameter of the target lesion or the disappearance of all target lesions for 4 weeks; PD was defined as an increase of at least 20% of the longest diameter of the target lesion, and SD indicated neither sufficient shrinkage to qualify for OR nor sufficient increase to qualify for PD.

Plasma samples were collected and centrifuged, and the upper layer was obtained and stored at −80°C until further use. Two clinical plasma samples were simultaneously obtained for the plasma proteome and drug susceptibility testing of CTCs. We used 60 plasma samples in proteomic analysis as quality control (QC) to evaluate the stability of our method.

### 5.2 Preparation of plasma

Plasma samples were obtained as described in our previous protocol [16]. The LH‐1808 Fully Automatic Liquid Handling Platform (AMTK) was used to automatically process 32 plasma samples simultaneously. In summary, 5 μL plasma was diluted 20 times with Tris‐HCl buffer (pH 8, Sigma, Germany) containing 10 mM Tris (2‐carboxyethyl) phosphine hydrochloride (TCEP, Sigma) and 50 mM 2‐chloroacetamide (CAA, Sigma). Samples were heated at 95°C for 10 min to denature the proteins. Next, a 20‐μL sample volume was auto‐pipetted followed with trypsin (Promega) digestion for 3 h at 37°C (protein:enzyme ratio = 50:1 m/m). The solution was then quenched by 48 μL of 0.1% (v/v) TFA. Finally, the digested peptides obtained were desalted and stored for −80°C until further analysis.

### 5.3 LC‐MS/MS analysis

The Exploris 240 mass spectrometer (Thermo Fisher Scientific) coupled with a Dionex UltiMate 3000 RSLCnano System (Thermo Fisher Scientific) was used for analysis. The peptides were loaded and separated with a 15 cm × 100 μm i.d. The C18 capillary column (1.9 μm, 120 Å) was used at a flow rate of 300 nL/min. FA (0.1%, v/v) in water, and 80% ACN was used in the mobile phases A and B for separation, respectively. The peptides were separated as follows: 4%–10% buffer B for 2 min, 10%–28% buffer B for 50 min, 28%–45% buffer B for 10 min, 45%–99% buffer B for 2 min, 99% buffer B for 10 min, and 99%–4% buffer B for 0.5 min. All MS spectra were acquired with a m/z 350–1550 with a mass resolution of 60,000 in data‐dependent analysis mode, in which the 12 most intense ions were selected for MS/MS scanning via higher‐energy collisional dissociation with 30% normalized collision energy. Tandem MS was acquired at a resolution of 15,000 and using an isolation window of 1.3 Da. The dynamic exclusion duration was set at 30 s.

### 5.4 ELISA

Plasma concentrations of SERPINA5, C4BPB, F5, AMBP, SELL, and PROZ were measured using ELISA kits (EK7383, EK19903, EK13890, EK11337, EK16554, and EK16276; Signalway Antibody) according to the manufacturer’s instructions.

### 5.5 Drug susceptibility testing of CTCs

Peripheral blood (7.5 mL) was centrifuged at 1600 g for 10 min, and the upper layer was discarded. Erythrocyte lysis buffer (Thermo Fisher Scientific) was added and samples were rotated for 15 min at 10 rpm followed by centrifugation at 500 *g* for 10 min. The precipitated cells were resuspended with glucose‐containing RPMI 1640 medium (Thermo Fisher Scientific) to prepare single cell suspensions. The CTCs were then isolated by FlowCell^TM^ cell sorter (Polaris Biology, China) based on differences in cell size, karyoplasmic ratio, and cell morphology. Using this method, 30–100 CTCs were captured from peripheral blood. However, leukocytes could also be isolated and were the main components.

The drug susceptibility of obtained CTCs was tested. The mixture generated by FlowCell^TM^ cell sorter was centrifuged at 500 *g* for 10 min, and the upper layer was discarded. The precipitated cells were resuspended with 10% FBS‐containing (Thermo Fisher Scientific) RPMI 1640 medium and inoculated into a 384‐well plate with 10,000 cells per well. Chemotherapy drugs were diluted into low (0.1 × IC50), medium (IC50), and high (10 × IC50) concentrations and added to each well with two replicates, and a control without chemotherapy drugs was set. The chemotherapy regimens were as follows: oxaliplatin/5‐fluorouracil (5‐FU), cis‐platinum/5‐FU, capecitabine/oxaliplatin, irinotecan/5‐FU, irinotecan/cis‐platinum/5‐FU, irinotecan/oxaliplatin/5‐FU, and raltitrexed/oxaliplatin. Cells were incubated for 24 h at 37°C under 5% CO_2_ and then cultured in glucose‐free RPMI 1640 medium with CD45 antibody (Cell Signaling Technology), 2‐NBDG (Thermo Fisher Scientific), and Hoechst 33,342 (Solarbio Life Science) for 4 h. Finally, the BackDrop Background Suppressor (Thermo Fisher Scientific) was added, and the plate was scanned with InvitrogenTM EVOSTM (Thermo Fisher Scientific). CTCs could be recognized according to cell morphology, CD45 antibody, and glucose metabolic analysis. We measured the fluorescence intensity of each well and compared it with the control to determine the glucose metabolic rate (GMR) for activity detection of CTCs. The GMR was an indicator of the survival of CTCs treated with chemotherapy, and a high GMR was associated with drug resistance.

$$\text{Chemosensitivity}\;\text{level}=\frac{100-\left(1-\frac{\text{GMR}1+\text{GMR}2+\text{GMR}3}{3}\right)*100}{50}$$

GMR1, GMR2, and GMR3 represented GMR at low, medium, and high concentrations of each chemotherapy regimen, respectively. Chemosensitivity levels would be <0.8, 0.8–1.0, 1.0–1.2, and >1.2, which indicated high sensitivity, moderate sensitivity, low sensitivity, and resistance, respectively. The higher the number, the stronger the drug resistance.

### 5.6 Data analysis

MS peak identification was processed using the Sequest HT node integrated within the Proteome Discoverer software (Version 2.5, Thermo Fisher Scientific) interrogating the human UniProt database (74,811 entries, downloaded in March 2020). For LFQ, MS data were processed using MaxQuant (Version 2.0.1.0) against the UniProt human protein database (70,956 entries, downloaded in July 2022). Variable protein modifications included methionine oxidation, acetylation of protein at N‐terminal. Fixed modification consisted of cysteine carbamidomethylation. Trypsin was established as a specific proteolytic enzyme with two missed cleavages. The peptide tolerance and the product ion tolerance were set at 4.5 and 20 ppm, respectively. At least one unique peptide was set for protein quantification. For peptide and protein identification, the false discovery rate cut‐offs were set at 0.01. A match between runs was selected. All other parameters were set to default values.

Protein intensities were quantile normalized using RStudio with the limma package. For proteomic data, proteins identified in more than 75% of the samples were included for further analysis, and missing values were imputed using normal distribution. SIMCA (version 14.1) was used for PCA analysis. Volcano plot, Pearson correlation, and KEGG and GO pathway analyses were performed using RStudio.

## AUTHOR CONTRIBUTIONS

Jingxin Yang and Jin Chen designed the manuscript. Jingxin Yang collected clinical information, performed ELISA, and drafted the manuscript. Jin Chen implemented the proteomic experiments, conducted data processing, and revised the manuscript. Luobin Zhang collected the clinical plasma samples. Xiaozhen Cui participated in automatic plasma proteome experiments. Fangming Zhou performed ELISA. Ruijun Tian and Ruilian Xu supervised the study.

## CONFLICT OF INTEREST STATEMENT

The authors Jingxin Yang, Jin Chen, Luobin Zhang, Fangming Zhou, Xiaozhen Cui, Ruijun Tian, and Ruilian Xu declare no conflict of interest.

## ETHICS STATEMENT

All procedures performed in these studies were in accordance with the ethical standards of the institution or practice at which the studies were conducted and with the 1964 Declaration of Helsinki and its later amendments or comparable ethical standards.

## Supporting information

Supporting Information S1

Table S1

Table S2

Table S3

Table S4

Table S5

Table S6

## Data Availability

Proteomic datasets in this study are available at iProX website with the dataset identifier PXD043974.
