# Supplementary material for: Plasma proteome profiling reveals biomarkers of chemotherapy resistance in patients with advanced colorectal cancer
Source: Quant Biol. 2024 Feb 14;12(2):215–24. doi: 10.1002/qub2.34 (PMC12806364; doi:10.1002/qub2.34)
Supplement: Supplementary file 1 — Supporting Information S1 [file QUB2-12-215-s003.docx]

Supplementary Material

Plasma proteome profiling reveals biomarkers of chemotherapy resistance in patients with advanced colorectal cancer

Jingxin Yang^1,†^, Jin Chen^2,3,†^, Luobin Zhang^4^, Fangming Zhou^1^, Xiaozhen Cui^3^, Ruijun Tian^3,*^, Ruilian Xu^4,*^

^1^Medical Genetic Center, Affiliated Shenzhen Maternity & Child Healthcare Hospital, Southern Medical University, Shenzhen, Guangdong, China

^2^Clinical Center for Molecular Diagnosis and Therapy, the Second Affiliated Hospital of Fujian Medical University, Quanzhou, Fujian, China

^3^Department of Chemistry and Research Center for Chemical Biology and Omics Analysis, School of Science, Southern University of Science and Technology, Shenzhen 518055, China

^4^Department of Oncology, Shenzhen People’s Hospital (The Second Clinical Medical College, Jinan University; The First Affiliated Hospital, Southern University of Science and Technology), Shenzhen, Guangdong, China

^†^These authors contributed equally to this work and share first authorship

*** Correspondence:**

Ruijun Tian

[tianrj@sustech.edu.cn](mailto:tianrj@sustech.edu.cn)

Ruilian Xu

[xuruilian2018@126.com](mailto:xuruilian2018@126.com)

**Contents**

Figure S1. The performances of QC samples during the plasma detection of 60 CRC patients. (A) LFQ intensities of five proteins covering nearly five orders of magnitude in QC samples. (B) LFQ intensities of all quantified proteins from QC samples.

Figure S2. The expression of 6 candidate proteins in QC to evaluate their stability in MS detection.

Figure S3. (A, B) The expression of F5 (A) and PROZ (B) proteins of patients with different chemotherapy outcomes detected by MS. (C, D) The spectra of unique peptides. (C) The fragment of peptide “GLLSGWAR” of protein PROZ. (D) The fragment of peptide “LLSLGAGEFK” of protein F5.

Figure S4. The expression of SERPINA5, C4BPB, SELL and AMBP proteins of patients with different chemotherapy outcomes detected by MS and their fragment spectra of unique peptides.

Table S1. Quantification of 5 technical workflow replicates.

Table S2. Detailed clinical information of 60 CRC patients.

Table S3. Quantification of plasma proteins from 60 CRC patients.

Table S4. The results of drug susceptibility testing of CTCs.

Table S5. The Pearson correlation between protein levels and chemotherapy resistances.

Table S6. Detailed clinical information of 79 CRC patients used in a validation cohort.


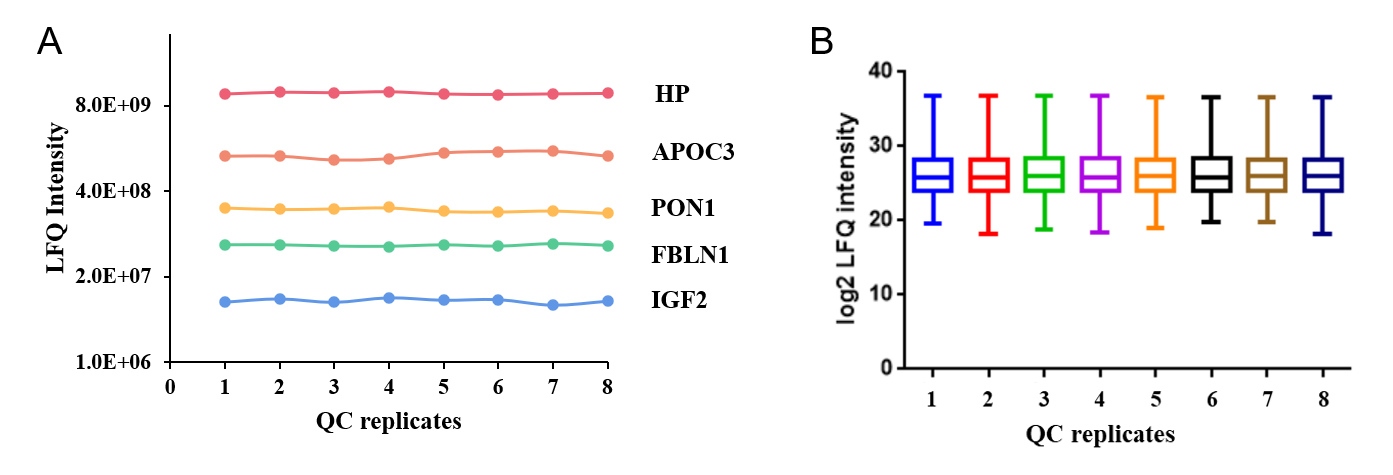


**Figure S1.** The performances of QC samples during the plasma detection of 60 CRC patients. (A) LFQ intensities of five proteins covering nearly five orders of magnitude in QC samples. (B) LFQ intensities of all quantified proteins from QC samples.

**
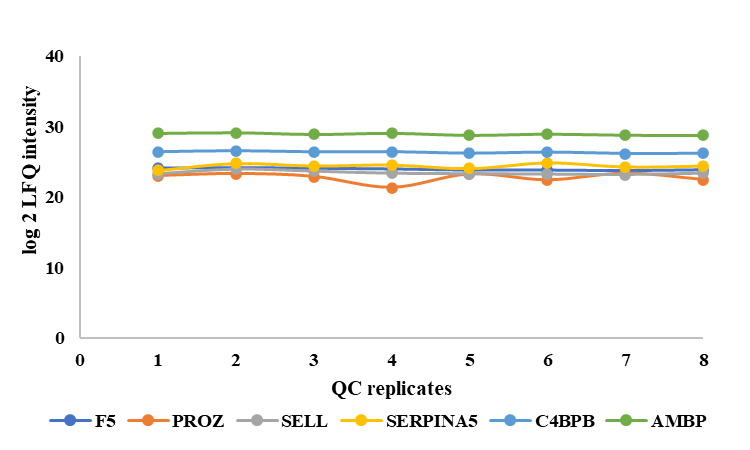
**

**Figure S2.** The expression of 6 candidate proteins in QC to evaluate their stability in MS detection.


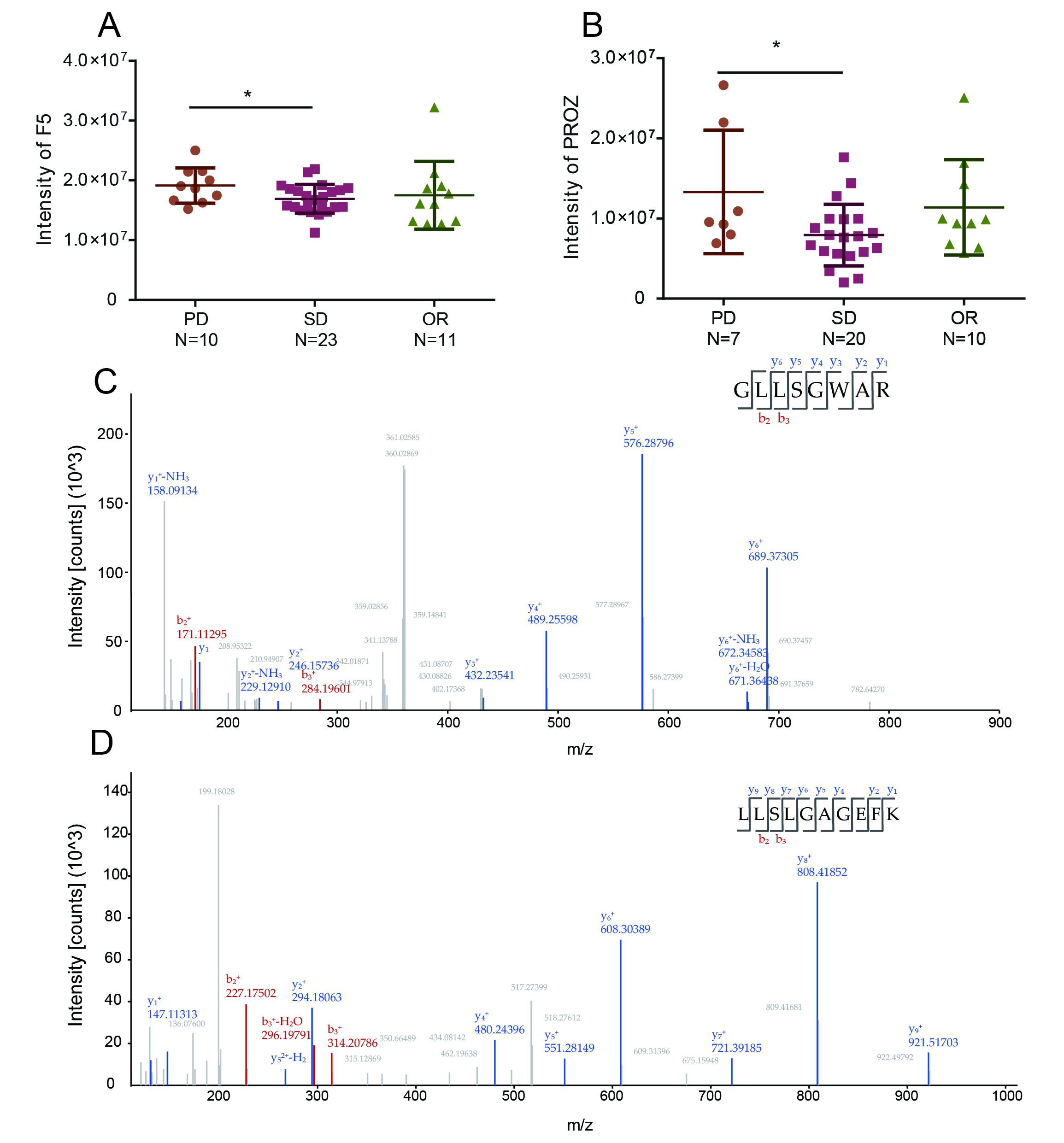


**Figure S3.** (A, B) The expression of F5 (A) and PROZ (B) proteins of patients with different chemotherapy outcomes detected by MS. (C, D) The spectra of unique peptides. (C) The fragment of peptide “GLLSGWAR” of protein PROZ. (D) The fragment of peptide “LLSLGAGEFK” of protein F5.


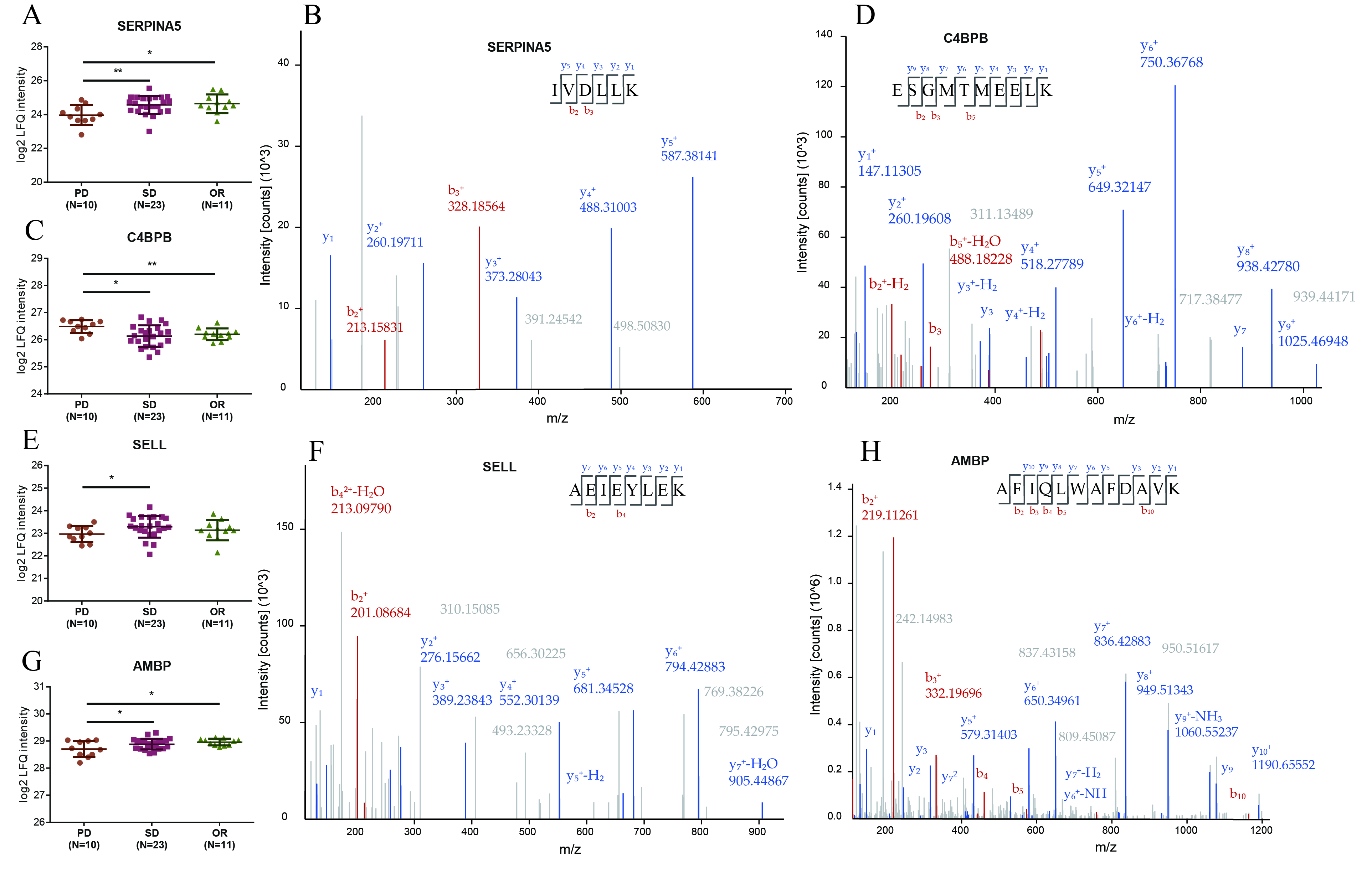


**Figure S4.** The expression of SERPINA5, C4BPB, SELL and AMBP proteins of patients with different chemotherapy outcomes detected by MS and their fragment spectra of unique peptides.
